# Activation of BK_Ca_ Channels in Zoledronic Acid-Induced Apoptosis of MDA-MB-231 Breast Cancer Cells

**DOI:** 10.1371/journal.pone.0037451

**Published:** 2012-05-24

**Authors:** Yu-Guang Ma, Wen-Chao Liu, Shuo Dong, Cheng Du, Xiao-Jun Wang, Jin-Sheng Li, Xiao-Ping Xie, Li Wu, Da-Chang Ma, Zhi-Bin Yu, Man-Jiang Xie

**Affiliations:** 1 Department of Clinical Oncology, Xijing Hospital, Fourth Military Medical University, Xi'an, China; 2 Department of Breast Disease, First Hospital of Lanzhou University, Lanzhou, China; 3 Department of Medicine, Baylor College of Medicine, Houston, United States of America; 4 Key Laboratory of Aerospace Medicine, Department of Aerospace Physiology, Fourth Military Medical University, Ministry of China, Xi'an, China; National Taiwan University Hospital, Taiwan

## Abstract

**Background:**

Zoledronic acid, one of the most potent nitrogen-containing biphosphonates, has been demonstrated to have direct anti-tumor and anti-metastatic properties in breast cancer *in vitro* and *in vivo*. In particular, tumor-cell apoptosis has been recognized to play an important role in the treatment of metastatic breast cancer with zoledronic acid. However, the precise mechanisms remain less clear. In the present study, we investigated the specific role of large conductance Ca^2+^-activated potassium (BK_Ca_) channel in zoledronic acid-induced apoptosis of estrogen receptor (ER)-negative MDA-MB-231 breast cancer cells.

**Methodology/Principal Findings:**

The action of zoledronic acid on BK_Ca_ channel was investigated by whole-cell and cell-attached patch clamp techniques. Cell apoptosis was assessed with immunocytochemistry, analysis of fragmented DNA by agarose gel electrophoresis, and flow cytometry assays. Cell proliferation was investigated by MTT test and immunocytochemistry. In addition, such findings were further confirmed with human embryonic kidney 293 (HEK293) cells which were transfected with functional BK_Ca_ α-subunit (*hSlo*α). Our results clearly indicated that zoledronic acid directly increased the activities of BK_Ca_ channels, and then activation of BK_Ca_ channel by zoledronic acid contributed to induce apoptosis in MDA-MB-231 cells. The possible mechanisms were associated with the elevated level of intracellular Ca^2+^ and a concomitant depolarization of mitochondrial membrane potential (Δψm) in MDA-MB-231 cells.

**Conclusions:**

Activation of BK_Ca_ channel was here shown to be a novel molecular pathway involved in zoledronic acid-induced apoptosis of MDA-MB-231 cells *in vitro*.

## Introduction

Breast cancer is the most common neoplasm in women and has a strong propensity to metastasize to bone. Most patients with advanced breast cancer frequently develop bone metastases characterized with the increased osteoclastic bone resorption, and at this stage, the disease associated with pain, fractures, and hypercalcemia is considered incurable [1]. More recently, multiple preclinical and early clinical studies have demonstrated that bisphosphonates are successfully established drugs that reduce the incidence of hypercalcaemia and skeletal morbidity in the treatment of breast cancer and bone metastasis [2]. The clinical potential of zoledronic acid, one of the most potent nitrogen-containing biphosphonates, is widely confirmed in the adjuvant and neoadjuvant settings of treatment for metastatic breast cancer [3], [4]. Zoledronic acid has been reported not only to inhibit osteoclast-mediated bone resorption, but also have direct anti-tumor and anti-metastatic properties in breast cancer *in vitro* and *in vivo* [4]. The primary mechanisms responsible for the direct anti-tumor activity of zoledronic acid may involve the inhibition of tumor-cell proliferation, the induction of tumor-cell apoptosis and autophagy, the prevention of tumor-cell invasion and adhesion in bone, the reduction of angiogenesis, and the stimulation of innate anti-cancer immunity [2], [4], [5]. In particular, tumor-cell apoptosis is an active, gene-regulated cell death, which has been considered to play a pivotal role in the treatment of breast cancer with zoledronic acid [2], [4], [5], [6]. However, the precise mechanisms by which zoledronic acid induces apoptosis in breast cancer cells remain to be determined [7].

Apoptosis is characterized by a distinct series of morphological and biochemical changes that result in cell shrinkage, DNA breakdown and phagocytic death. There are at least two regulatory pathways that can lead to apoptosis [8]. The extrinsic pathway (or the death receptor pathway) involves the binding of apoptotic signals to a death receptor and subsequent caspase activation. The intrinsic pathway (or the mitochondrial pathway) is triggered by the depolarization of mitochondrial membrane or DNA damage, which relies on the disruption of the mitochondrial membrane. Recently, many studies suggested that large conductance Ca^2+^-activated K^+^ (BK_Ca_) channels are involved in the regulation of apoptosis. BK_Ca_ channels are ubiquitously present in most human cells and play an essential role in the regulation of basic cellular processes [9], [10]. The basic functional unit of BK_Ca_ channel is the pore forming α-subunit encoded by a single gene, *Sloα* or *KCNMA1*. BK_Ca_ channels are activated by membrane potential, intracellular Ca^2+^, and phosphorylation. Activation of BK_Ca_ channel hyperpolarizes the membrane potential and deactivates the voltage-dependent Ca^2+^ channels (VDCCs), which leads to a reduction in intracellular Ca^2+^ concentration. In excitable cells, such as vascular smooth muscle cells (VSMCs), it is well known that BK_Ca_ channels contribute to the regulation of vascular tone in a negative feedback manner which limits VSMCs depolarization and prevents vasospasm [11]. Recently, activation of BK_Ca_ channel has also been reported to be involved in the regulation of apoptosis besides its electrophysiological function in vascular relaxation [12], [13]. In contrast, functions of the BK_Ca_ channel in non-excitable cells are somewhat enigmatic. Previous studies have implicated a role for the BK_Ca_ channel in the progression of several malignant tumors, including metastatic breast cancer [14], [15], [16], osteosarcoma [17], prostate cancer [18], [19], colorectal carcinogenesis [20], ovarian cancer [21], and glioma [22], [23], [24], [25]. In particular, it has been demonstrated that BK_Ca_ channels are highly expressed in various established human breast cancer cell lines, such as MCF-7, MDA-MB-231, MDA-MB-468, MDA-MB-435s, and MDA-MB-361 [26], [27]. However, the role of BK_Ca_ channel in phenomena related to breast cancer is still controversial. For example, activation of BK_Ca_ channel has been described to be involved in the proliferation [14], [16], [26], migration, and invasion [15] of breast cancer cells. In contrast, some work also suggested that BK_Ca_ channel might have no roles in the controlling growth of breast cancer cells because the specific BK_Ca_ channel blockers [charybdotoxin or iberiotoxin (IBTX)] did not have any effect on cell proliferation [27]. However, to date there have been no studies addressing the possibility of BK_Ca_ channel involvement in the regulation of apoptosis in human breast cancer cells.

**Figure 1:**
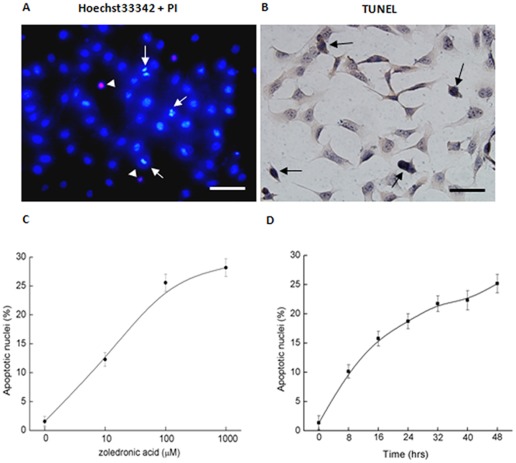
Apoptotic effects of zoledronic acid on MDA-MB-231 cells. (A) Nuclear condensation and fragmentation of single cell were shown with representative Hoechst33342 + PI double-staining. Arrow indicated the apoptotic nuclei and arrowhead indicated the necrotic nuclei. (B) Positive transferase-mediated nick-end labeling (TUNEL)-stained nuclei were used in some experiments. (C) Dose-dependent curve is shown for the apoptotic effects of zoledronic acid concentration (0–1000 μM) for 48 h. (D) Time-dependent curve is shown for the apoptotic effects of 100 μM zoledronic acid (D). Values are means ± SE; n = 40∼80 fields of cells in 8 independent experiments for each data point. (Scale bar in A and B: 25 μm)

The purpose of this study was to examine the specific role of BK_Ca_ channel in zoledronic acid-induced apoptosis of breast cancer cells. The ER-negative MDA-MB-231 cell lines were chosen for the experiment. The action of zoledronic acid on BK_Ca_ channel was investigated by whole-cell and cell-attached patch clamp techniques. Cell apoptosis was assessed with immunocytochemistry, analysis of fragmented DNA by agarose gel electrophoresis, and flow cytometry assays. Cell proliferation was investigated by MTT test and immunocytochemistry. In addition, such findings were further confirmed from human embryonic kidney 293 (HEK293) cells which were transfected with functional BK_Ca_ α-subunit (*hSlo*α). Finally, intracellular Ca^2+^ and mitochondrial membrane potential (Δψm) in MDA-MB-231 cells were also examined to investigate the possible mechanisms. Evidence obtained in the present study suggests that zoledronic acid directly increased the activities of BK_Ca_ channels, and then activation of BK_Ca_ channel by zoledronic acid contributed to induce apoptosis in ER-negative MDA-MB-231 breast cancer cells. The possible mechanisms were associated with the elevated level of intracellular Ca^2+^ with a concomitant depolarization of Δψm in MDA-MB-231 cells. Our data are the first to show involvement of BK_Ca_ channel activation in zoledronic acid-induced apoptosis of of ER-negative breast cancer cells *in vitro*.

## Results

### Zoledronic acid induced apoptosis in MDA-MB-231 breast cancer cells

Treatment with zoledronic acid for 48 h significantly induced apoptosis in MDA-MB-231 cells with its typical characteristics of nuclear condensed or fragmented (Figure 1A) as well as its positive-staining for TUNEL (Figure 1B). Apoptotic MDA-MB-231 cells in response to zoledronic acid were dose-dependent with an EC_50_ of ∼17 μM (Figure 1C) and time-dependent (Figure 1D). The apoptotic effects of zoledronic acid seemed to be maximized at the concentration of 100∼1000 μM. As compared with vehicle controls, the administration of 100 μM zoledronic acid for 48 h induced a lower rate of necrotic cell death (5.56±2.65% *versus* 1.23±0.6%). However, the administration of 1000 μM zoledronic acid for 48 h not only induced a significant increase in apoptotic rates (28.16±1.54% *versus* 1.55±0.84%, Figure 1C), but also induced a higher rate of necrotic cell death (red fluorescent chromatin, 25.12±1.87% *versus* 1.23±0.6%), which indicated the cytotoxic effects of zoledronic acid at high concentration. Therefore, 100 μM zoledronic acid was selected as the working concentration to induce apoptosis (Figure 1C). In addition, administration of 100 μM zoledronic acid for only 8 h could significantly increased the percentage of apoptotic nuclei from 1.36±1.20% to 10.13±1.15%, and the apoptotic effects appeared to be maximized (to 25.17±1.57%) at about 48 h during our observation (Figure 1D). Our results that zoledronic acid significantly induced apoptosis of MDA-MB-231 breast cancer cells are consistent with a previous report [6].

**Figure 2:**
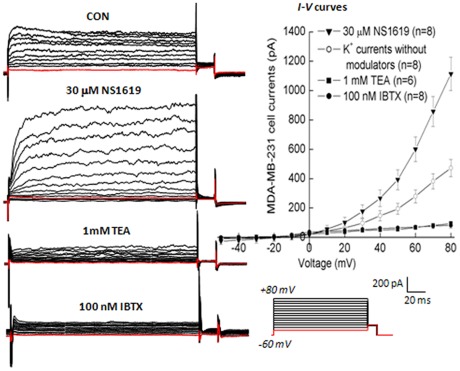
Representative families of whole-cell outward currents recorded from MDA-MB-231 cells without BK_Ca_ channel modulators, in the presence of agonist NS1619, or antagonist TEA and IBTX to the bath solution. In whole-cell configuration, membrane potential was held at −60 mV and stepped at 15-s intervals to potentials between −50 mV and +80 mV in 10-mV increments for 200 ms and then held at +30 mV for 20 ms. Average data are shown by means ± SE with the number of cells recorded in parentheses in *I-V* curves which are shown for each condition in the right panel.

### Augmenting effects of zoledronic acid on BK_Ca_ channel activity in MDA-MB-231 breast cancer cells

Whole-cell currents in MDA-MB-231 cells showed time- and voltage-dependent outward-currents (the left panel of Figure 2). Acute extracellular application of 30 μM NS1619, the specific agonist of BK_Ca_ channel, significantly and reversibly amplified the whole-cell currents by 2-fold as compared with the control at the testing potential of +60 mV. Subsequently, extracellular application of 1 mM tetraethylammonium (TEA, the nonselective BK_Ca_ inhibitor) or 100 nM IBTX (the specific BK_Ca_ blocker) significantly reduced the outward-current amplitudes and diminished the current noise associated with higher positive command potentials. NS1619 has been reported to be highly selective for activating BK_Ca_ channels through α-subunit [10]. TEA predominantly blocks BK_Ca_ channel currents at doses of ≤1 mM [32]. The current-voltage relationship (*I-V*) curves were generated by plotting currents against command potentials (the right panel of Figure 2). These results clearly identified the properties of BK_Ca_ currents recorded from MDA-MB-231 cells, which were in agreement with previous report [27].

**Figure 3:**
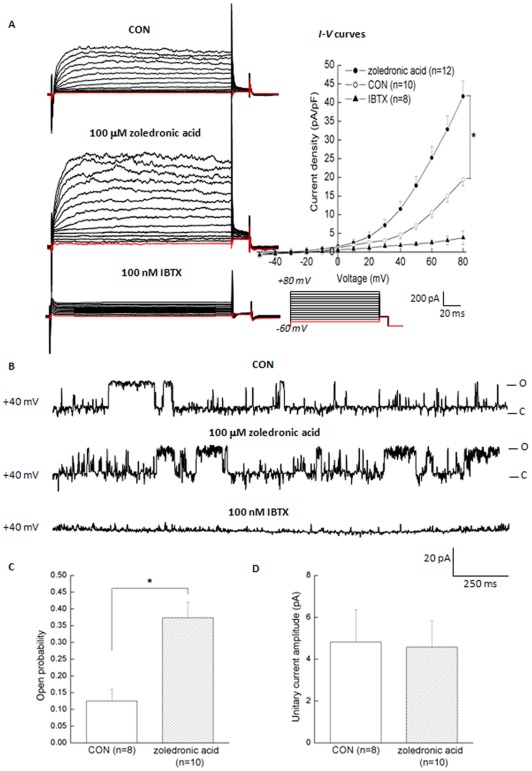
Actions of zoledronic acid on BK_Ca_ whole-cell and single-channel activities in MDA-MB-231 cells. (A) Representative families of BK_Ca_ whole-cell currents in MDA-MB-231 cells before and after application of 100 μM zoledronic acid. Application of 100 nM IBTX in the continued presence of zoledronic acid significantly inhibited BK_Ca_ whole-cell currents. The mean *I-V* curves were further expressed in terms of current densities. (B) Representative traces of BK_Ca_ single-channel currents in cell-attached patches before and after application of 100 μM zoledronic acid to the bath solution. In cell-attached patches, the membrane voltage was held at +40 mV and the Ca^2+^ concentration in the bath fluid was 1.98 mM. (C) Plots of open probability (*Po*) and unitary current amplitude (*Am*) in BK_Ca_ channels were shown against membrane potentials. Values are means ± SE with the number of cells examined is in parentheses. *P<0.05 as compared with the control by ANOVA. (O: open state; C: close state)

Experiments were then undertaken to determine the action of zoledronic acid on BK_Ca_ channels in MDA-MB-231 cells. Acute addition of 100 μM zoledronic acid to the bathing solution led to a significant increase in whole-cell outward currents (the left panel of Figure 3A). For example, at the testing potential of +60 mV, zoledronic acid significantly increased outward-current densities by 2.5-fold as compared with the control. However, acute application of 100 nM IBTX in the continued presence of zoledronic acid significantly reduced whole-cell currents. The mean *I-V* relationships were further expressed in terms of current densities (the right panel of Figure 3A). Additional experiments were performed to determine whether zoledronic acid affects BK_Ca_ single-channel activity in MDA-MB-231 cells. The representative traces of single-channel current were obtained at +40 mV from cell-attached patches before and after the addition of 100 μM zoledronic acid to the bath solution (Figure 3B). Zoledronic acid significantly increased open probability (*Po*) by 3-fold at +40 mV (Figure 3C) but had no effects on unitary current amplitude (*Am*) (Figure 3D). Taken together, these observations clearly indicated that zoledronic acid increased the whole-cell and single-channel activities of BK_Ca_ channel recorded from MDA-MB-231 cells, which has not been reported previously in breast cancer cells.

**Figure 4:**
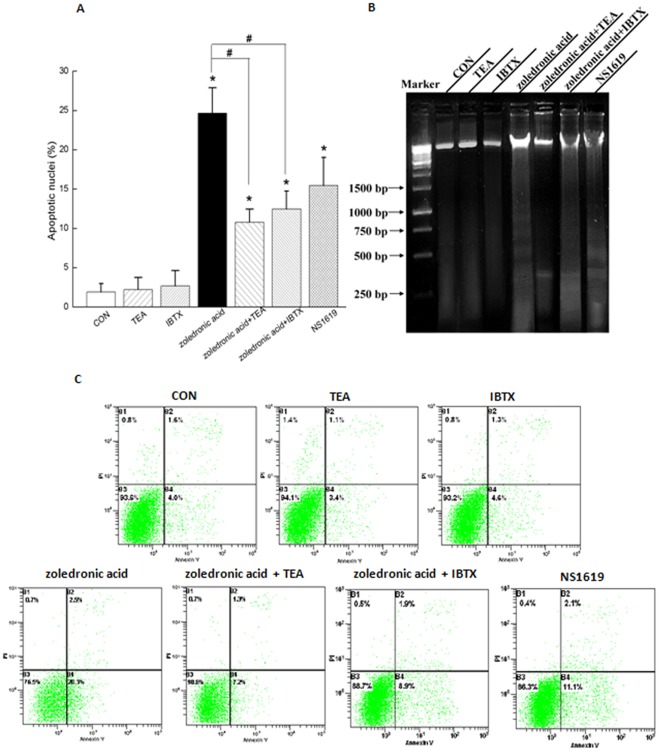
Activation of BK_Ca_ channel by 100 **μM zoledronic acid induced apoptosis of MDA-MB-231 cells.** (A) Summarized data from Hoechst33342 + PI double-staining showed the percentage of apoptotic rates in MDA-MB-231 cells treated with different conditions. Values are means ± SE. n =  40∼60 fields of cells in 8 independent experiments for each data point. Apoptosis of MDA-MB-231 cells were analyzed by agarose gel electrophoresis (B) and Annexin V + PI double-binding from cytometry (C). In the dot plots of Annexin V + PI double-binding, viable cells (Annexin V-low/ PI-low) are found in the lower left quadrant, apoptotic cells (Annexin V-high/ PI-low) in the lower right, postapoptotic secondary necrotic cells (Annexin V-high/ PI-high) in the upper right and primary necrotic cells (Annexin V-low PI-high) in the upper left. Numbers in each quadrant are percentage of cells they contain. Images in (B) and (C) represent 3 and 5 independent experiments, respectively. *P<0.05 as compared with the control; #*P*<0.05 as compared with the treatment of zoledronic acid.

### Activation of BK_Ca_ channel by zoledronic acid induced apoptosis in MDA-MB-231 breast cancer cells

To investigate the role of BK_Ca_ channels in zoledronic acid induced-apoptosis, Hoechst33342 + PI double-staining (Figure 4A), analysis of fragmented DNA (Figure 4B), and flow cytometry assays (Figure 4C) were used to evaluate apoptosis of MDA-MB-231 breast cancer cells. Treatment with 100 μM zoledronic acid for 48 h significantly caused about 24.65±3.25% cells to undergo apoptosis (Figure 4A). However, application of BK_Ca_ channel blocker (1 mM TEA or 100 nM IBTX) to the culture medium in the presence of zoledronic acid for 48 h could partially reverse zoledronic acid-induced apoptosis to 10.77±1.69% or 12.46±2.29%, respectively. In contrast, treatment with 1 mM TEA or 100 nM IBTX for 48 h alone did not induce significant apoptosis as compared with the control, respectively. Treatment with the specific agonist of BK_Ca_ channel (30 μM NS1619) for 48 h alone induced apoptosis to 15.44±3.57%, similar to the apoptotic effects of zoledronic acid (Figure 4A). To independently verify apoptosis qualitatively, fragmented DNA was analyzed by agarose gel electrophoresis, which is considered to be a biochemical hallmark for apoptosis. These characteristic changes associated with apoptosis are due to the activation of a family of intracellular caspases [12]. Treatment with 100 μM zoledronic acid or 30 μM NS1619 for 48 h could form a detectable ladder of multiples of 180–200 bp associated with the DNA fragmentation pattern in MDA-MB-231 cells (Figure 4B). However, application of 1 mM TEA or 100 nM IBTX to the culture medium in the presence of zoledronic acid for 48 h could also induce a detectable ladder but much fainter when compared to the treatment with zoledronic acid, which suggested that TEA or IBTX could partially reverse the apoptotic effects of zoledronic acid (Figure 4B). Lastly, we performed flow-cytometric analyses (Annexin V + PI double-binding) to confirm and quantify the induction of apoptosis in MDA-MB-231 cells. Treatment with 100 μM zoledronic acid or 30 μM NS1619 for 48 h significantly induced apoptosis in MDA-MB-231 breast cancer cells (Figure 4C, lower right quadrants). However, TEA or IBTX could partially reverse zoledronic acid-induced apoptosis. These observations from Annexin V + PI double-binding (Figure 4C) were in accordance with results from Hoechst33342 + PI double-staining (Figure 4A) or analysis of fragmented DNA (Figure 4B). These findings obviously indicated that activation of BK_Ca_ channel by zoledronic acid induced apoptosis in MDA-MB-231 breast cancer cells.

**Figure 5:**
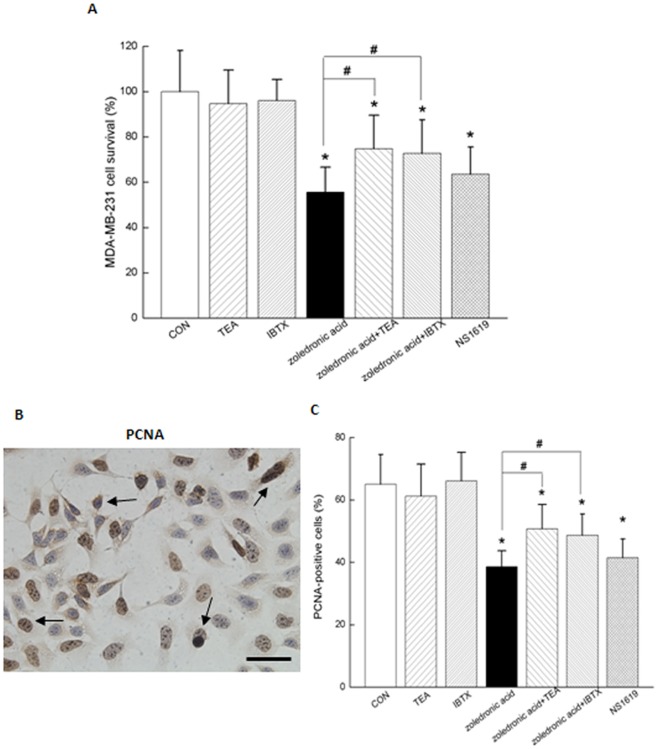
Activation of BK_Ca_ channel by 100 μM zoledronic acid suppressed proliferation of MDA-MB-231 cells. (A) Summarized data from MTT assays showed global cell viabilities in MDA-MB-231 cells treated with different conditions. Values are means ± SE in 6 independent experiments for each data point. (B) Representative proliferating cell nuclear antigen (PCNA)-positive nuclei were shown with arrow indication. (C) Summarized data from PCNA staining showed the percentage of proliferative rates in MDA-MB-231 cells treated with different conditions. Values are means ± SE; n =  30∼50 fields of cells in 5 independent experiments for each data point. *P<0.05 as compared with the control; #*P*<0.05 as compared with the treatment of zoledronic acid. (Scale bar in B: 25 μm).

### Activation of BK_Ca_ channel by zoledronic acid suppressed proliferation of MDA-MB-231 breast cancer cells

To evaluate the role of BK_Ca_ channel in zoledronic acid-inhibited cell growth, MTT assays (Figure 5A) and PCNA-staining (Figure 5B and Figure 5C) were used to assess cell viability and proliferation in MDA-MB-231 breast cancer cells. Treatment with 100 μM zoledronic acid or 30 μM NS1619 for 48 h not only exhibited a strong inhibitory action on cell survival (Figure 5A), but also significantly decreased the percentage of PCNA-positive cells as compared with the control (Figure 5B and Figure 5C). As a marker of cell proliferation, PCNA is associated with rapidly dividing cells. In the present study, NS1619 significantly reduced the proliferation of MDA-MB-231 cells, which was consistent with previous report [27]. However, TEA or IBTX, the blockers of BK_Ca_ channel, could partially reverse the anti-proliferative effects of zoledronic acid in MDA-MB-231 cells. In contrast, TEA or IBTX alone did not change cell viability and proliferation in MDA-MB-231 cells. These observations indicated that activation of BK_Ca_ channel by zoledronic acid significantly attenuated the proliferation of MDA-MB-231 cells.

**Figure 6:**
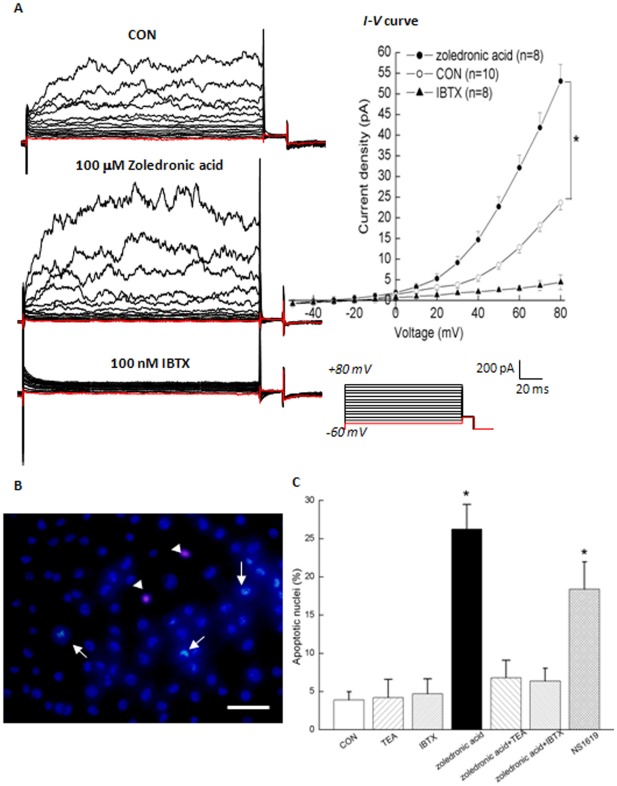
Activation of cloned BK_Ca_ channel by zoledronic acid induced apoptosis in HEK-*hSlo*α cells. (A) Representative families of BK_Ca_ whole-cell currents in HEK-*hSlo*α cells before and after application of 100 μM zoledronic acid. Application of 100 nM IBTX in the continued presence of zoledronic acid significantly inhibited cloned BK_Ca_ whole-cell currents. The mean *I-V* curves were further expressed in terms of current densities. (B) Representative Hoechst33342 + PI double-staining showed the apoptotic nuclei with arrow indications and necrotic nuclei with arrowhead indication in HEK-*hSlo*α cells. (C) Summarized data showed the percentage of apoptotic rates in HEK-*Slo*α cells treated with different conditions. Values are means ± SE; n = 20∼30 fields of cells in 5 independent experiments for each data point. *P<0.05 as compared with control. (Scale bar in B: 25 μm).

**Figure 7:**
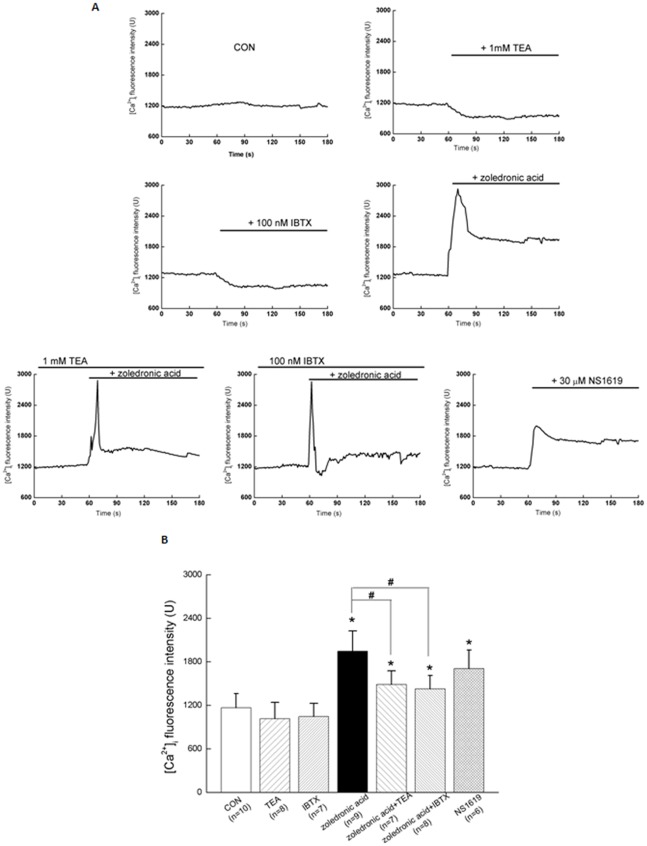
Activation of BK_Ca_ channel by zoledronic acid elevated the level of intracellular Ca^2+^ fluorescence intensity in MDA-MB-231 cells. (A) Representative Fluo-3/AM fluorescence intensities recorded in MDA-MB-231 cells before and after applications of TEA, IBTX, zoledronic acid, and NS1619. (B) Summarized data showed the average changes of Fluo-3/AM fluorescence intensities in MDA-MB-231 cells treatment with different conditions during 120 ∼180 s. Values are means ± SE with the number of cells recorded in parentheses. *P<0.05 as compared with the control; #*P*<0.05 as compared with the treatment of zoledronic acid.

### Activation of cloned BK_Ca_ channels by zoledronic acid induced apoptosis in transfected HEK293 cells

It has been demonstrated that endogenous currents are generally small and there are almost no endogenous BK_Ca_ currents in native HEK293 cells [28], [29]. Therefore, HEK293 cells were used to investigate the exogenous BK_Ca_ channels in isolation by removing other types of channel currents which can potentially contaminate BK_Ca_ currents in native MDA-MB-231 cells. Cloned BK_Ca_ α-subunit (*hSlo*α) were transiently transfected into HEK293 cells. *I-V* curves clearly identified that activities of cloned BK_Ca_ channel were augmented by acute application of 100 μM zoledronic acid and blocked by extracellular application of 100 nM IBTX in HEK-*hSlo*α cells (Figure 6A). Double-staining of Hoechst33342 + PI (Figure 6B and Figure 6C) showed that treatment with 100 μM zoledronic acid or 30 μM NS1619 for 24 h significantly induced 26.24±3.25% or 18.38±3.57% apoptosis of HEK-*hSlo*α cells as compared with the control (4.18±2.39%). However, the blocker of BK_Ca_ channel, 1 mM TEA or 100 nM IBTX could completely reverse zoledronic acid-induced apoptosis in HEK-*hSlo*α cells. In contrast, treatment with 1 mM TEA or 100 nM IBTX did not significantly induce apoptosis of HEK-*hSlo*α cells as compared with the control. In another experiment, we also found that zoledronic acid had no apoptotic effects in nontransfected HEK293 cells (4.51±0.65% in zoledronic acid *vs.* 3.45±1.39% in the control). These results suggested that zoledronic acid-induced apoptosis of HEK-*hSlo*α cells was mainly due to its activation of cloned BK_Ca_ channels and BK_Ca_ α-subunit may be a target for the action of zoledronic acid.

### Activation of BK_Ca_ channel in zoledronic acid-induced apoptosis was associated with the elevated level of cytosolic free Ca^2+^ and the depolarized mitochondrial membrane potential (Δψm) of MDA-MB-231 breast cancer cells

**Figure 8:**
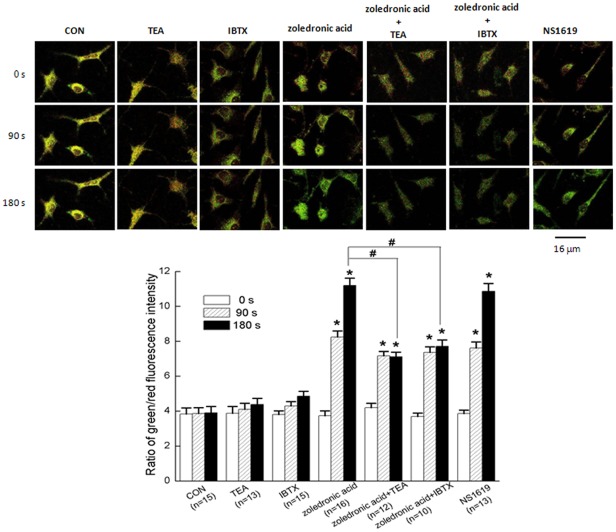
Activation of BK_Ca_ channel by zoledronic acid depolarized mitochondrial membrane potentials (Δψm) of MDA-MB-231 cells. (A) Representative fluorescent images of MDA-MB-231 cells stained with JC-1 probe in different groups (Scale bar: 16 μm). (B) Quantitative analysis of the shift of mitochondrial orange-red fluorescence in different groups at 0 s, 90 s, and 180 s, respectively. The average fluorescence intensities are expressed as the ratio of green to red fluorescence. An increase in the bar indicated a shift in the fluorescence ratio correlating with an increase in mitochondrial depolarization. Values are means ± SE with the number of cells recorded in parentheses. *P<0.05 as compared with the control; #*P*<0.05 as compared with the treatment of zoledronic acid at 90 s and 180 s.

To evaluate the possible mechanisms, the intracellular free Ca^2+^ ([Ca^2+^]_i_) and mitochondrial membrane potential (Δψm) of MDA-MB-231 cells were measured before and after applications of TEA, IBTX, zoledronic acid, and NS1619, respectively. The present data showed that 100 μM zoledronic acid evoked a transient peak increase of [Ca^2+^]_i_ (at 60 ∼80 s) and was followed by a sustained increase in [Ca^2+^]_i_ (at 120 ∼180 s) that was above the basal values in the continued presence of zoledronic acid (Figure 7A). Compared to the control, zoledronic acid significantly elevated the level of [Ca^2+^]_i_ by 66.7% in MDA-MB-231 cells (Figure 7B), which were consistent with previous report that zoledronic acid could increase [Ca^2+^]_i_ in human osteosarcoma cells probably owing to the release of Ca^2+^ from intracellular stores [34]. Furthermore, as compared with the control, zoledronic acid also induced a stronger mitochondrial depolarization of MDA-MB-231 cells indicated with obvious green fluorescence at 90 s and 180 s of scanning duration (Figure 8), which were in accordance with previous report that zoledronic acid gradually induces a decrease of Δψm in MCF-7 breast cancer cells and RPMI 8226 myeloma cells [35]. In addition, 30 μM NS1619 also significantly also evoked a significant augmentation of [Ca^2+^]_i_ by ∼46.2% and a stronger depolarization of Δψm as compared with the control. However, 1 mM TEA or 100 nM IBTX could partially reverse the effects of zoledronic acid on [Ca^2+^]_i_ (Figure 7) and depolarization of Δψm (Figure 8) in MDA-MB-231 cells, respectively. In contrast, only TEA or IBTX showed a reduced (but not significant) induction of [Ca^2+^]_i_ (Figure 7) and did not significantly change the Δψm (Figure 8). These results suggested activation of BK_Ca_ channel in zoledronic acid-induced apoptosis was associated with elevated levels of cytosolic free Ca^2+^ and the depolarization of Δψm in MDA-MB-231 breast cancer cells.

## Discussion

The present study makes two novel findings. Firstly, zoledronic acid directly activated BK_Ca_ channel, and then activation of BK_Ca_ channels by zoledronic acid was involved in initiating apoptosis of ER-negative MDA-MB-231 breast cancer cells *in vitro*. Such conclusions were further confirmed from HEK293 cells transfected with cloned BK_Ca_ α-subunit. Secondly, the possible mechanisms of activation of BK_Ca_ channels in zoledronic acid-induced apoptosis were associated with the increased intracellular Ca^2+^ and a concomitant depolarization of mitochondrial membrane potential in MDA-MB-231 breast cancer cells. Our study provided a novel molecular pathway whereby activation of BK_Ca_ channel by zoledronic acid induced apoptosis of ER-negative breast cancer cells *in vitro*.

Bisphosphonates are synthetic derivates of endogenous pyrophosphates in which the central atom of oxygen has been replaced by a carbon atom. *In vivo*, bisphosphonates bind strongly to hydroxyapatite on the bone mineral surface with high affinity and are preferentially delivered to sites of increased bone formation or resorption [2], [4]. Zoledronic acid, a cyclic bisphosphonate of the third generation, contains a nitrogen atom in an imidazole ring. It has been considered that zoledronic acid is the most potent anti-resorptive compounds to lower the serum calcium concentration (hypercalcaemia) and reduce the excessive bone loss and fracture risk associated with malignant skeletal diseases [2], [3], [4]. In addition, emerging evidence also suggests a beneficial preventive treatment of zoledronic acid in patients with early stages of breast cancer without bone metastases [36], [37]. Laboratory studies suggest that zoledronic acid can also directly induce important anti-tumour effects in breast cancer by inhibiting cell adhesion and invasive potential, suppressing cell proliferation, and inducing tumor-cell apoptosis [2], [4], [6]. In particular, it has been recognized that tumor-apoptosis plays an important role in the treatment of breast cancer with zoledronic acid [4], [5]. However, the underlying mechanisms of action by which zoledronic acid induces apoptosis in breast cancer cells remain less clear. One possible mechanism is that zoledronic acid inhibits farnesyl pyrophosphate synthase (FPPS), a key enzyme in the mevalonate pathway, which results in decreased isoprenoid production and protein prenylation, and then affects the intracellular signaling pathways, such as failure to activate small GTPases (Ras and Rho), down-regulation of α_v_β_3_/α_v_β_5_ integrins, and releasing cytochrome C into the cytosol with subsequent activation of the caspase cascade [4], [37]. Another possible mechanism is that inhibition of FPPS also causes accumulation of ATP analogues (Apppi), which can directly induce apoptosis by disrupting mitochondrial ATP/ADP translocase [4], [35]. In agreement with previous reports [1], [6], we observed that zoledronic acid significantly increased apoptosis in a concentration and time dependent manner in MDA-MB-231 cells (Figure 1). It is considered that the net apoptotic effects of zoledronic acid on breast cancer cells are determined by a variety of pathways [3], [4], [5]. Therefore, we explored the novel mechanisms pathways to explain the possible mechanisms of zoledronic acid induced-apoptosis.

In the present study, we demonstrated that zoledronic acid significantly increased activities of BK_Ca_ channel (Figure 3), and then, activation of BK_Ca_ channels by zoledronic acid significantly induced apoptosis (Figure 4) and suppressed cell proliferation (Figure 5) in MDA-MB-231 breast cancer cells. However, blocking BK_Ca_ channels by 1 mM TEA or 100 nM IBTX could partially reverse apoptotic effects and anti-proliferative effects of zoledronic acid in MDA-MB-231 breast cancer cells. Such conclusions were further confirmed from the cloned BK_Ca_ channels in HEK-*hSlo*α cells (Figure 6). These observations strictly indicated that activation of BK_Ca_ channel by zoledronic acid induced apoptosis in ER-negative MDA-MB-231 breast cancer cells.

To date, it is notable that a controversial role for BK_Ca_ channels has been reported in carcinogenesis *in vivo* and *in vitro*. Some studies have suggested that BK_Ca_ channels contributed to the high proliferative or invasive potential in a number of malignant cell lines, such as non-metastatic (MCF-7) breast cancer cells [14], [26], brain-specific metastatic (MDA-MB-361) breast cancer cells [15], human prostate cancer [19], colorectal carcinogenesis [20], glioma [24], [25]. However, more recent publications put forward the opposite idea that BK_Ca_ channels are not required for the proliferation in glioma [22] or breast cancer cells [27]. What is more, BK_Ca_ channels have been reported to exhibit anti-proliferative and anti-tumorogenic properties in osteosarcoma cells [17], ovarian cancer cells [21], and glioma cells [23]. In the present study, we reported that BK_Ca_ channel directly participated in the regulation of zoledronic acid-induced apoptosis in human MDA-MB-231 breast cancer cells (Figure 4 and Figure 6). The apparent discrepancy between these studies may be explained by distinct roles of BK_Ca_ channels in different cancer cell lines and different tumor microenvironment [17], [22].

It is believed that different breast cancer cell lines have distinct properties, so the results of BK_Ca_ channels from MDA-MB-231 breast cancer cell line *in vitro* may not be generalized to the other breast cancer cell lines. For example, we also observed the role of BK_Ca_ channels in zoledronic acid-induced apoptosis in MCF-7 cells. Unlike apoptosis in MDA-MB-231 cells, zoledronic acid-induced apoptosis in MCF-7 cells was resistant to BK_Ca_ channels blockers. Interestingly, when MCF-7 cells were treated with the ER inhibitor ICI182780, zoledronic acid-sensitive apoptosis regained its partial sensitivity to BK_Ca_ channel inhibitors (data not shown). It remains unclear why these differences exist and required further investigations.

Taken together, our work reported *in vitro* evidence that zoledronic acid directly increased the activities of BK_Ca_ channel, and then activation of BK_Ca_ channel by zoledronic acid may be partially responsible for zoledronic acid induced-apoptosis in MD-MBA-231 breast cancer cells. Such conclusion was further supported from HEK293 cells transfected with cloned BK_Ca_ channels. The possible mechanisms were associated with the elevation of intracellular Ca^2+^ and the depolarization of Δψm in MD-MBA-231 breast cancer cells. Therefore, the new recognition of zoledronic acid on BK_Ca_ channels may offer opportunities to develop a novel pharmacological approach in the treatment of breast cancer.

## Materials and Methods

### Chemicals and reagents

Zoledronic acid (Zometa, Novartis Pharmaceuticals Corp, Shwewiz AG, Switzerland) was provided as the hydrated disodium salt and dissolved in dH_2_O. Unless otherwise stated, chemicals used in this study were obtained from Sigma Chemical Company (St. Louis, MO).

### Cell culture and transfection

MDA-MB-231 breast cancer cell line and HEK293 cells were purchased from the American Type Culture Collection (ATCC; Manassas, VA). MDA-MB-231 and HEK293 cells were cultured at 37°C and 5% CO_2_ in DMEM (Gibco BRL, Grand Island, NY) supplemented with 5% and 10% FBS (HyClone, Logan, UT), respectively. The *hSlo*α cDNA plasmid was cloned in the expression vector pIRES (Clontech Laboratories, Palo Alto, CA; pIRES-*hSlo*α). The transfection was performed by Lipofectamine^TM^ 2000 (Invitrogen, Carlsbad, CA) as described before [28], [29]. To diminish the influence of serum on cell growth, cells were cultured in 1% FBS +1% Insulin-Transferrin-Selenium (ITS) during the pharmacological experiments.

### Electrophysiological measurements

Whole-cell and single-channel of BK_Ca_ currents were recorded with an amplifier (CEZ-2300, Nihon Kohden Co., Tokyo, Japan) and a version interface (Axon Instruments, Foster City, CA) as reported previously [13], [30], [31]. Whole-cell BK_Ca_ currents were recorded with the conventional voltage clamp configuration. Current densities were obtained by normalizing currents to the cell membrane capacitance (Cm). The extracellular (bath) solution contained 135 mM NaCl, 5.0 mM KCl, 1.8 mM CaCl_2_, 1.0 mM MgCl_2_, 10 mM HEPES, 10 mM glucose, and 5.0 mM 4-aminopyridine (4-AP), equilibrated with 95% O_2_ and 5% CO_2_ at pH 7.4 adjusted by NaOH. 4-AP in the bath solution was used to exclude the interference from voltage-dependent K^+^ (K_V_) channel currents [32]. The internal (pipette) solution contained 50 mM KCl, 70 mM K-Asp, 8.0 mM NaCl, 2.0 mM MgCl_2_, 1.0 mM Na_2_ATP, 0.5 mM GTP, 10 mM HEPES, 1.0 mM CaCl_2_, 2.0 mM EGTA equilibrated with 95% O_2_ and 5% CO_2_ at pH 7.2 titrated with KOH.

Single-channel currents of BK_Ca_ were recorded in cell-attached membrane patches. The pipette (external) solution contained 40 mM K-Asp, 100 mM KCl, 1.0 mM CaCl_2_, 10 mM HEPES equilibrated with 95% O_2_ and 5% CO_2_ at pH 7.4 titrated with KOH. The bath solution contained 100 mM K-Asp, 40 mM KCl, 10 mM HEPES, 2.0 mM EGTA, 1.98 mM CaCl_2_ equilibrated with 95% O_2_ and 5% CO_2_ at pH 7.4 titrated with KOH.

### Apoptosis assays

#### Morphological assessment of apoptotic cells

Two fluorescent nuclear binding dyes, Hoechst33342 and propidium iodine (PI) were added to the culture medium to a final concentration of 5 μg/ml. Cells were evaluated by fluorescence microscopy according to the following grading system: normal nuclei (blue chromatin with organized structure), apoptotic cells (bright fluorescent chromatin which is highly condensed or fragmented), and necrotic cells (red fluorescent chromatin) as described previously [28], [29]. The apoptotic index was calculated as (number of apoptotic cells/total cells counted) ×100%. Scoring was done blindly.

In some experiments, Terminal deoxynucleotidyl transferase-mediated dUTP Nick End labeling (TUNEL) assay was performed to detect apoptosis with the *in situ* DeadEnd^TM^ Colorimetric TUNEL System (Promega, USA) [13], [28], [29]. Briefly, fragmented DNA was nick end-labeled with a mixture of Terminal Deoxynucleotidyl Transferase, Recombinant (rTdT) enzyme and Biotinylated Nucleotide Mix in an Equilibration Buffer. The reaction was stopped and horseradish peroxidase-labeled streptavidin (Streptavidin HRP) was then bound to the biotinylated fragmented DNA, which was detected using the peroxidase substrate, hydrogen peroxide, and the stable chromogen, diaminobenzidine (DAB).

#### Analysis of fragmented DNA by agarose gel electrophoresis

DNA fragmentation assay was performed using the previously described methods with some revisions [28], [29]. Briefly, cells were lysed with buffer containing 10 mM Tris-HCl, 10 mM EDTA, and 0.5% Triton X-100 at pH 7.6 titrated with Tris base (pH 8.0). The fragmented DNA was extracted with phenol/chloroform/isopropanol (25:24:1, v/v) and then extracted again with chloroform/isopropanol (24:1, v/v). The DNA fragments were separated by 2% agarose gel electrophoresis.

#### Flow cytometry assays for apoptotic cell death

Cells were harvested and incubated with FITC-labeled Annexin V (Merck Biosciences, Bad Soden, Germany) and the DNA-binding dye, PI. The percentage of apoptotic and necrotic cells were determined by flow cytometry (FACScalibur, Becton Dickinson Immunocytometry Systems, San Jose, CA, USA). Five parallel samples were measured and ten thousand events were analyzed using the Cell Quest Pro software (Becton Dickinson Immuzoledronic acidcytometry System).

### Proliferation assays

#### MTT assays for cell viability

After incubation with MTT (5 mg/ml), cells were treated with DMSO to dissolve the purple formazan crystals formed [28], [29]. The optical density was recorded using a micro-plate reader (μQuant, Bio-Tek Instruments, Inc., USA) at 490 nm. The cell viability was calculated by dividing the optical density of samples with the optical density of solvent control.

#### Proliferating cell nuclear antigen (PCNA) staining

As previous described [29], fixed cells were incubated with a 1∶50 dilution of the mouse anti-human PCNA monoclonal antibody (Santa Cruz, USA) and then incubated with the biotinylated goat anti-mouse IgG in a dilution of 1∶200 (Santa Cruz, USA). Finally, the ready-to-use streptavidin-horseradish-peroxidase complex conjugated avidin biotin complex (1∶100) (Santa Cruz, USA) was applied and the peroxidase colour reaction was started by incubation with 0.04% (w/v) DAB. The proliferative index was calculated as (number of proliferative cells/total cells counted) ×100%. Scoring was done blindly.

#### Measurement of intracellular Ca^2+^

As previous described [29], [31], [33], cells were incubated with Fluo-3-acetoxymethyl ester (Fluo-3/AM, Invitrogen, USA) in a concentration of 5 μM. During continuously scanning with a laser confocal microscope (Olympus FV1000, Japan), different pharmacological reagents were added to the cell and a period of 3 min was recorded. When the mean fluorescence became constant, the average fluorescence intensity was used to indicate the changes of intracellular Ca^2+^ during scanning period of 120∼180 s. To avoid any laser-induced changes in Ca^2+^ signaling, each cell was scanned only once.

### Mitochondrial membrane potential (Δψm) assays

As previously described [29], 5,5′,6,6′-tetrachloro-1,1′,3,3′-tetraethylbenzimidazolcarbocyanine (JC-1, Molecular Probes, USA) is a kind of potentiometric dye which exhibits membrane potential dependent-loss as J-aggregates (polarized mitochondria) transition to JC-1 monomers (depolarized mitochondria) as indicated by the fluorescence emission shift from red to green. Cells were loaded with JC-1 in a final concentration of 5 μg/ml and then scanned. The Δψm was monitored by determining the relative amounts of dual emissions from mitochondrial JC-1 monomers to J-aggregates. Mitochondrial depolarization was indicated by an increase in the green/red fluorescence intensity ratio.

### Statistical Analysis

Data are expressed as means ± SE. A one-way ANOVA was used to determine the significant differences in the experiments. Post Hoc tests were used to determine where statistically significant differences were located in apoptotic and proliferative rates among the groups (Tukey's test). A value of *P*<0.05 was considered to be statistically significant.
